# Mesenchymal Stem/Progenitor Cells and Their Derivates in Tissue Regeneration

**DOI:** 10.3390/ijms23126652

**Published:** 2022-06-15

**Authors:** Aleksandra Klimczak

**Affiliations:** Laboratory of Biology of Stem and Neoplastic Cells, Hirszfeld Institute of Immunology and Experimental Therapy, Polish Academy of Sciences, R. Weigla 12, 53-114 Wroclaw, Poland; aleksandra.klimczak@hirszfeld.pl

## 1. Background

Mesenchymal stem/stromal cells (MSC) have been extensively studied over the last 30 years in the context of their regenerative and immunomodulatory activities for potential application in regenerative medicine. However, the hopes observed in the early stage of research to use MSCs as a potential remedy for many diseases are still not fully satisfied. Currently, the effectiveness of MSCs therapies has been verified in 1430 clinical trials, registered at the www.clinicaltrials.gov (as of 24 May 2022), concerning the supportive therapy of cells and organs transplantations; osteoarticular, autoimmune, cardiovascular and neurodegenerative diseases; and recently, on COVID-19 treatment. However, there are still many issues that need to be explored.

Cells bearing the criteria of MSC characteristics are isolated from different tissues of the adult organism, including: bone marrow, adipose tissue, skin, skeletal muscle, synovial membrane, tendon, bone, brain, liver, lungs, and others, and from perinatal tissues such as cord blood or the umbilical cord [1]. Many studies indicate the dependence of the effectiveness of the MSC therapy of a given disease on the tissue source of their origin. In self-renewing organs, tissue-specific progenitor cells reside in specific niches that constitute the microenvironment in which they survive. Our earlier studies documented similarities and differences between MSCs originating from different tissues in terms on their surface markers and their regenerative potential in organs where they reside, as well as their multipotential ability to differentiate into other lineages [2]. However, MSCs also possess immunomodulatory, anti-inflammatory, anti-apoptotic, and proangiogenic properties due to the secretion of broad range of bioactive factors, and thus, they may modulate in the local environment, leading to tissue regeneration [3]. The therapeutic effect of the MSC secretome is mediated mainly through the growth factors and cytokines secreted by parental cells. Proteomic analysis of MSC-conditioned media (MSC-CM) revealed the presence of several hundred proteins involved in angiogenesis, the regulation of apoptosis, and immune response [4]. Moreover, MSCs also release various bioactive molecules in a form of extracellular vesicles (EVs), exosomes (Exo), and microvesicles (MVs), which act on neighboring cells and tissues by exerting paracrine effects.

All the papers comprising this Special Issue share the latest data and opinions and can help to explain the pro-regenerative potential of MSCs and their derivatives in tissue regeneration.

## 2. Bone Regeneration, Tissue Engineering and MSC Secretome

Tissue engineering in regenerative medicine is currently a focus especially for bone regeneration. To create scaffolds biocompatible with MSCs is very challenging and requires specific strategies to induce osteoinductivity. The SmartBone^®^ (SB) is a biohybrid bone substitute, proposed as a CE-labeled class III medical device, for bone regeneration in reconstructive surgeries in different skeletal regions for oral, maxillofacial, orthopedic, and oncologic needs. To improve osteoinductivity, the SB scaffolds were loaded with lyosecretome composed of a freeze-dried formulation of adipose-tissue-derived MSC-secretome containing proteins and extracellular vesicles (EVs) [5]. The work performed by Bari et al. demonstrated that lyosecretome-loaded SmartBone^®^ (SBlyo) can improve the osteoinductive potency of the adipose-tissue-derived stromal vascular fraction (SVF), and this effect is apparently related with the osteogenic factors present in the lyosecretome, such as fibronectin, alpha-2-macroglobulin, apolipoprotein A, and TGF-β. The combination of a standardized and ready-off-the-shelf MSC-secretome powder with tissue engineering strategies is a promising cell-free based therapy approach which may result in next-generation osteoinductive scaffolds that can improve quality and time of bone regeneration.

Working on the osteogenic potential of scaffold supported with dental-pulp-derived MSCs (DPSCs) or BMSCs, Shiu and co-workers assessed the bone healing capacities of synthetic bone-grafting materials (micro- and macro-biphasic calcium phosphate (MBCP)) and xenografts (Bio-Oss) in a rabbit calvarial bone defect model [6]. The results showed that bone grafting materials and the bioactivity of MSCs can synergistically enhance new bone formation; however, autogenic bone still is the gold standard in bone regeneration.

Molecular and transcriptomic background of osteogenesis of hADSCs was studied by Kim and co-workers in two-dimensional (2D) and three-dimensional (3D) culture conditions [7]. The comprehensive transcriptome expression profiles during osteogenic differentiation of hADSCs under different culture conditions revealed that the genes related to bone development and bone remodeling processes were overexpressed in the 3D culture condition. This research could have potential therapeutic applications, such as in designing molecular factors for various bone regeneration scaffolds.

Dental stem cells can be isolated from various dental soft tissues, including human dental pulp (hDPSCs) and share MSC characteristics similar to MSC of BM-origin. Current knowledge on the DPSCs-derived secretome, in a form of conditioned medium and/or EVs, as a potential candidate in the regeneration of bone, cartilage, and neural injuries is presented in the review by Bar research group [8]. The regenerative effect of human hDPSC-derived secretome used in experimental studies is mainly accomplish by regulating neuroprotective, anti-inflammatory, antiapoptotic, and angiogenic processes through paracrine factors released by human dental-MSC.

## 3. Solid Organs Dysfunction and MSCs Secretome

Applications of extracellular vesicles (EVs) are also widely studied as a therapeutic strategy in solid organs disease. Bruno’s research group investigated the therapeutic effect of EVs originated from liver-derived MSC-like population in the mouse experimental model of renal ischemia and reperfusion injury (IRI), that subsequently is leading to the development of chronic kidney disease [9]. The results showed that EV treatment immediately after induction of renal IRI decreased the development of interstitial fibrosis by modulating the expression levels of specific genes that are involved in fibrosis as confirmed at the histological and molecular levels.

Chronic liver injuries lead to liver fibrosis and then to end-stage of liver dysfunction. Extensive studies on liver regeneration using MSCs and their secretome are currently the focus in preventing liver fibrosis, and this is a subject of the review presented by Nazarie (Ignat) et al. [10]. Experimental studies showed that the use of MSCs-EVs as treatment for liver fibrosis may be more effective than MSCs therapy, as they can go through biological barriers and transport their anti-fibrotic cargo to target cells. Moreover, MSC-EVs’ cargo can be modified in order to deliver specific proteins of miRNAs with anti-fibrotic properties.

Chronic obstructive pulmonary disease is often associated with cigarette smoking exposure. Chen and co-workers studied the protective effects of adipose-derived stem cell-conditioned medium (ADSC-CM) against cigarette-smoke-extract-induced cell death and epithelial–mesenchymal transition (EMT) on human lung cancer and non-cancerous epithelial cells model [11]. ADSC-CM effectively inhibited EMT induced by cigarette smoke extract and was able to reverse the gradual loss of epithelial marker expression associated with TGF-β1 treatment.

Interesting insights on tissue regeneration has been introduced by Dörnen and Dittmar in the review paper on the role of MSCs and cell fusion in tissue regeneration. MSCs could restore injured tissue by adopting the phenotype of damaged tissue, e.g., liver, neuronal, muscle, and intestinal cells through cell fusion. However, in case of cancer cells, cell fusion leads to an enhanced heterogeneity including a high genetic and epigenetic variability and is associated with increased tumorigenicity, metastasis, and therapy resistance [12].

Therapeutic effects of MSCs are accomplished by variety of bioactive mediators, such as growth factors, cytokines, and extracellular vesicles that have immunosuppressive, anti-apoptotic, anti-fibrotic, angiogenic, and anti-inflammatory properties. These bioactive factors released during cells culture also constitute MSCs secretome in a form of conditioned media. Studies by Chugh et al. showed that the BMP-2 secreted by BM-hMSC can treat hyperandrogenemia by suppressing steroidogenesis and gene expression involved in androgen synthesis in the H295R cell line and in the ovaries of a letrozole-induced polycystic ovary syndrome in the mouse model [13].

## 4. Angiogenic Dysfunction and MSCs Secretome

The curative potential of AT-MSC on wound healing has been extensively studied by many research groups. Kraskiewicz et al. propose a cell-free therapy by using hydrogel loaded with a secretome from the established human AT-MSC to facilitate the translation of cell therapy bioactive factors into clinical application [14]. The incorporation of AT-MSC bioactive factors into collagen hydrogel exert pro-proliferative and pro-angiogenic activity tested in an in vitro wound model. Moreover, AT-MSC conditioned medium revealed antimicrobial activity; therefore, in combination with the hydrogel, it has potential to be used as advanced wound-healing dressing acting as a reservoir of a therapeutic factor which is being released in a sustained manner.

Adipose-tissue-derived MSCs, alongside bone marrow, are the most commonly used source of mesenchymal stem/stromal cells due to their good accessibility. The review by Krawczenko and Klimczak is focused on the role of mesenchymal stem/stromal cells of adipose tissue origin (AT-MSCs) and their secretome in the regulation of damaged tissue regeneration [15]. Special attention is given to the contribution of AT-MSCs and their derivatives, EVs, and conditioned medium to angiogenic processes mainly in the context of angiogenic dysfunction. AT-MSCs secretome serve as a carrier of bioactive factors supporting vessel, muscular, neural, and cutaneous repair and regeneration.

The impaired blood–brain barrier (BBB) is responsible for secondary brain injuries of the CNS after stroke, traumatic brain injury, epilepsy, and other damages. Following ischemia, BBB disruption initiates a series of adverse events causing vasogenic edema, neuroinflammatory response, and cell death, resulting in long-term adverse effects. MSCs and their derivatives are considered as potential candidates for post-stroke cell therapy. The review by Do et al. highlights the molecular mechanisms of BBB preservation by MSCs therapy following ischemic stroke, related to the maintenance of BBB integrity [16].

## 5. Biological and Functional Assessment of MSCs and Secretome

Studies performed by Navakauskiene’s research group are focused on endometrial stem cells as an attractive target for studies on infertility of unexplained origin in women [11]. Menstrual-blood-origin stem cells are morphologically and functionally similar to cells derived from the endometrium. They found that the induction of the menstrual stem cells for differentiation into epithelial cells increased expression of genes related to decidualization (PRL, ESR, IGFBP, and FOXO1) and angiogenesis (HIF1, VEGFR2, and VEGFR3). The authors suggest that stem cells of endometrial origin isolated from menstrual blood could be an alternative source of cellular therapy for the improvement of endometrium function for females with diagnosed unexplained infertility treatment [11].

Vasicek presented a study about the possibility to analyze the secretome of rabbit AT-MSCs by using a large-scale cytokine array designed for human samples [17].

## 6. Conclusions

The presented original studies and review analyses will expand our understanding of MSCs and their bioactive factors in the form of conditioned medium or MVs and provide new insights into its potential biological activity for tissue regeneration. Studies documented that MSC derivatives carry miRNA and proteins that support regenerative processes and can be used as cell-free therapies in potential clinical applications.
